# Impact of smoking Behavior on cognitive functioning in persons at risk for psychosis and healthy controls: A longitudinal study

**DOI:** 10.1192/j.eurpsy.2021.2233

**Published:** 2021-09-21

**Authors:** Heleen S. van der Heijden, Frederike Schirmbeck, Matthew J. Kempton, Mark van der Gaag, Kelly Allot, Barnaby Nelson, Stephan Ruhrmann, Lieuwe de Haan, Jentien M Vermeulen, Eu-Gei High Risk Study

**Affiliations:** 1 Department of Psychiatry, Amsterdam UMC (location AMC), Amsterdam, The Netherlands; 2 Arkin Institute for Mental Health, Amsterdam, The Netherlands; 3 Department of Psychosis Studies, Institute of Psychiatry, Psychology & Neuroscience, King’s College London, United Kingdom; 4 Psychosis Research Institute, Parnassia Group, Hague, The Netherlands; 5 Department of Clinical Psychology, Vrije Universiteit, Amsterdam, The Netherlands; 6 Centre for Youth Mental Health, University of Melbourne, Melbourne, Australia; 7 Orygen, The National Centre of Excellence in Youth Mental Health, Parkville, Australia; 8 Department of Psychiatry and Psychotherapy, Faculty of Medicine and University Hospital, University of Cologne, Cologne, Germany

**Keywords:** clinical high risk, cognition, nicotine, psychosis, smoking

## Abstract

**Background:**

The high prevalence of smoking in individuals who are at ultra-high risk (UHR) for psychosis is well known and moderate cognitive deficits have also been found in UHR. However, the association between smoking and cognition in UHR is unknown and longitudinal studies are lacking.

**Method:**

A cohort study with 330 UHR individuals and 66 controls was conducted, as part of the European network of national schizophrenia networks studying gene–environment interactions (EU-GEI). At baseline and after 6, 12, and 24 months, smoking behavior was assessed with the Composite International Diagnostic Interview and cognitive functioning with a comprehensive test battery. Linear mixed-effects analyses were used to examine the multicross-sectional and prospective associations between (change in) smoking behavior and cognitive functioning, accounting for confounding variables.

**Results:**

At baseline, 53% of UHR and 27% of controls smoked tobacco. Smoking UHR and controls did not significantly differ from nonsmoking counterparts on the tested cognitive domains (speed of processing, attention/vigilance, working memory, verbal learning, or reasoning/problem solving) across different assessment times. Neither smoking cessation nor initiation was associated with a significant change in cognitive functioning in UHR.

**Conclusions:**

No associations were found between smoking and cognitive impairment in UHR nor in controls. However, the fact that one in every two UHR individuals report daily use of tobacco is alarming. Our data suggest that UHR have fewer cognitive impairments and higher smoking cessation rates compared to patients with first-episode psychosis found in literature. Implications to promote smoking cessation in the UHR stage need further investigation.

## Introduction

In patients with psychotic disorders, smoking is highly prevalent [1]. Moreover, smoking prevalence in individuals who are at risk for psychosis—also known as ultra-high risk (UHR) individuals—exceeds three times the prevalence rate found in age-matched controls [2,3]. In individuals with a psychotic disorder and also in UHR individuals, a widespread impairment of neurocognitive functioning has been observed [4]. Deficits in verbal learning, visual memory, processing speed, attention/vigilance, and intelligence contribute to the prediction of later transition to psychotic disorder [4].

A modifiable factor that is probably related to cognition is smoking [5]. *Acute* nicotine administration is associated with beneficial effects on cognition by increasing activity in several brain regions [6]. However, the relationship between nicotine administration and cognitive functioning is complex and not completely disentangled [7]. Distinguishing between cognitive effects of *acute* or *chronic* nicotine use is important: concerning the latter, a substantial body of literature in both the general population as in patients with psychosis have found worse cognitive functioning in chronic nicotine users. For example, memory performance accuracy was poorer in adolescent smokers than in nonsmokers [8]. Furthermore, cognitive impairments were more severe with earlier age of onset of smoking. Another study [9] showed that elderly smokers are at a higher risk for cognitive impairment and that this risk increases with the duration and intensity of smoking and subsides with time after smoking cessation. These results are in line with findings from a recent meta-analysis in patients with established psychosis, where chronic smoking was related to impairments across several cognitive domains [10]. Additionally, a longitudinal study of patients with psychosis and their unaffected siblings found that smoking cessation was associated with an improvement in processing speed [11]. There is a lack of longitudinal studies evaluating the association between nicotine (both acute as well as chronic) and cognitive functioning in UHR individuals. So far, no longitudinal and only one cross-sectional study [12] with a small sample size have been conducted. This is unfortunate, given the presence of cognitive deficits [13], their impact on functional outcome [14], and the high smoking prevalence [15] in this vulnerable group.

Therefore, the aim of the current study is to examine the association between smoking and cognitive performance on several domains across different assessment times in an UHR population and a healthy control group. Based on the findings from populations with established psychosis [10] and unaffected siblings [11], we hypothesized that smoking status or the number of cigarettes smoked per day would be negatively associated with cognitive functioning in UHR as well as in healthy controls. Furthermore, we aimed to explore whether cessation of smoking was associated with improvements in cognitive functioning.

## Methods

### Study design

This study was performed within the European network of national schizophrenia networks studying gene–environment Interactions (EU-GEI) cohort [16,17]. EU-GEI is a multicenter, prospective naturalistic study conducted between May 1, 2010 and April 30, 2015 and consisted of a baseline measurement and three follow-up points (at 6 months, 1 year, and 2 years). The aim of EU-GEI is the identification of clinical, genetic, and environmental interactions in the development, severity, and course of (subclinical) psychotic disorders in participants and their families. The EU-GEI study was conducted in accordance with the Declaration of Helsinki and the protocol of the study was approved by the institutional review boards of all study sites.

### Participants

Individuals were recruited from 11 early detection centers (Amsterdam, Den Haag, Vienna, Basel, Cologne, Melbourne, Copenhagen, Paris, Barcelona, Sao Paulo, and London). Further details are also described in Supplementary Material S1. Participants were aged between 14 and 45 years (mean age: 22 years). Participants were included in the study if they met at least one of the three UHR criteria as defined by the *Comprehensive Assessment of At-risk Mental State* (CAARMS) [18]: (a) Vulnerability Group: a first-degree relative with a psychotic disorder or diagnosed with schizotypal personality disorder in combination with a significant drop in functioning during at least 1 month in the previous year; (b) Attenuated Psychotic Symptoms (APS) Group: the presence of subthreshold positive psychotic symptoms for at least 1 month during the past year; or (c) Brief Limited Intermittent Psychotic Symptoms (BLIPS) Group: an episode of frank psychotic symptoms that lasted no longer than 1 week, which abated spontaneously. Exclusion criteria were a prior experience of a psychotic episode of more than 1 week as determined by the CAARMS [18] or an intelligence quotient (IQ) below 60. Control participants were recruited from four different sites (London, Amsterdam, Den Haag, and Melbourne). Exclusion criteria for controls were similar to those for UHR participants. Additionally, controls were excluded if they met the criteria for an ARMS status as defined by the CAARMS [18]. Individuals were analyzed for whom complete data for smoking status was available at baseline. Assessments at 6 months were scarce in most inclusion sites, however, not due to any patient-specific reasons, as this time point was introduced later in the study. EU-GEI researchers with various backgrounds (research assistants, psychologists, psychiatrists, nurses, and PhD students) were extensively trained to increase inter-rater reliability (IRR). All researchers achieved high IRR scores before permitted to perform assessments [16,19]. All participants provided written informed consent following a full explanation of the study.

### Assessment instruments

#### Smoking behavior

We used the *Composite International Diagnostic Interview* (CIDI) to assess detailed information on smoking behavior which has previously been found to be reliable in a cross-cultural trial [20]. Smokers were defined as people who smoked daily for at least 1 month over the past 12 months. Participants were asked how many cigarettes they smoked per day in the time frame they smoked the most during the last/past year.

#### Cognitive measurements

Each participant was assessed on cognitive performance on baseline, 6 months, 1 year, and 2 years in accordance with the Measurement and Treatment Research to Improve Cognition in Schizophrenia Consensus Cognitive Battery [21]. Cognitive performance included the following domains and tasks:

1. *Speed of processing* as measured with the Trail Making Test [22] part A.
2. *Attention/vigilance* as measured with the Digit Span Forward, a subtest of the third version of the Wechsler Memory Scale (WMS-III) [23].
3. *Working memory* as measured with the Digit Span Backward, a subtest of the WMS-III.
4. *Verbal learning* as measured with the Rey Auditory Verbal Learning Test (RAVLT) [24], including a component of immediate recall (trial 1–5) and delayed recall (trial 7).
5. *Cognitive flexibility* (reasoning and problem solving) as measured with the Trail Making Test part B.

#### Assessment of covariates

Age and gender were a priori selected covariates. Additionally, socioeconomic status (including social functioning, educational level, and work), cannabis use, childhood trauma, and psychiatric medication (including antipsychotics, antidepressants, and anxiolytics) were also selected as confounders, and psychopathology were selected as covariates as they have been found associated with both smoking and cognition in individuals with psychosis [11,25–27]. The general level of social functioning was scored using the disability scale of the *Global Assessment of Functioning* (GAF) [28]. Educational level was defined as total years in education. IQ was assessed with the 15-min version [29] of the Wechsler Adult Intelligence Scales third editions [30], but was not included as a covariate considering the overlap with years in education. Current employment was divided into two subgroups: no paid work and student or paid work. The *Cannabis Experience Questionnaire* was administered to assess information regarding the current and lifetime use of cannabis (yes or no). *The Childhood Trauma Questionnaire* was used to explore whether participants had experienced childhood trauma, which has been found to be a valid retrospective assessment instrument [31]. A total score of all five subscales (emotional abuse, physical abuse, sexual abuse, emotional neglect, and physical neglect) was computed as a covariate for the analyses. Severity of symptoms was measured using the CAARMS [18] and the following subscales were included in the analyses: general symptoms (in which the majority of items measure affective symptoms), positive, and negative symptoms.

### Statistical analyses

We used SPSS Statistics version 26 (Chicago, IL) for all analyses. Baseline differences in demographic characteristics and outcomes between smokers and nonsmoking UHR individuals were assessed using independent *t* tests and Mann–Whitney *U* tests for numerical variables and Pearson Chi-square tests for categorical variables. To test the first hypothesis, linear mixed models were applied to assess associations between smoking status, the number of cigarettes smoked per day, and cognitive performance scores across different assessment times over a period of 2 years (multicross-sectional). UHR individuals and controls were included in the analyses if data on the outcome variable of interest was available for at least one time point (baseline, 6 months, 1 year, or 2 years) as mixed modeling allowed us to calculate valid estimates under the assumption of missing at random. Little’s MCAR test [32] appeared to be nonsignificant indicating that data were missing at least at random, which was in line with visual inspection of missing data patterns on baseline. Models were fitted using maximum likelihood estimation [33,34] and continuous variables were centered [35] to improve model performance and interpretability [33]. As outcome variables, raw scores for each cognitive domain were used. Visual inspection of residual plots of all cognitive measurements revealed no deviations from normality, with exception of the cognitive performance scores on the Trail Making Test (parts A and B). In the first model, smoking status (yes/no), time, age, and gender were entered as fixed effects. In case simple models showed significant results, all other a priori selected covariates were planned to be added en bloc: GAF, education, work, cannabis, trauma scores, and medication. In all models, subjects were added as random intercept and time was added as random slope. In order to investigate a dose–response relationship, linear mixed effect models were run replacing smoking status by the number of cigarettes smoked per day. To answer the second research question regarding the effect of change in smoking behavior, we compared smoking behavior at 1-year follow-up to baseline as well as at 2-year follow-up to 1-year follow-up. We categorized four UHR subgroups between assessments: individuals who never smoked, individuals who continued smoking, individuals who were able to quit, and individuals who started smoking. Change scores between assessments were calculated for all outcome variables. In the first set of mixed model analyses, individuals who never smoked were compared with individuals who started smoking. In a later set, we compared individuals who continued to smoke with individuals who were able to quit. Additionally, linear mixed effect models with change in the number of cigarettes smoked per day as independent variable were performed. Similar fixed and random effects as previously mentioned were entered. No longitudinal analyses regarding change in smoking status and/or cigarettes were performed in healthy controls due to a lack of power (Supplementary Material S5b). In all mixed model analyses, *p* values were calculated by the Satterthwaite method which has been evaluated in REML-fitted models and produced the most acceptable type I error rates in mixed-effects models [36]. Given the six outcome variables in five independent cognitive domains, Bonferroni correction was used to minimize the risk of type I errors. Therefore, the two-tailed significance threshold was set at 0.008 (0.05/6).

## Results

### Sample characteristics

**Figure 1.**
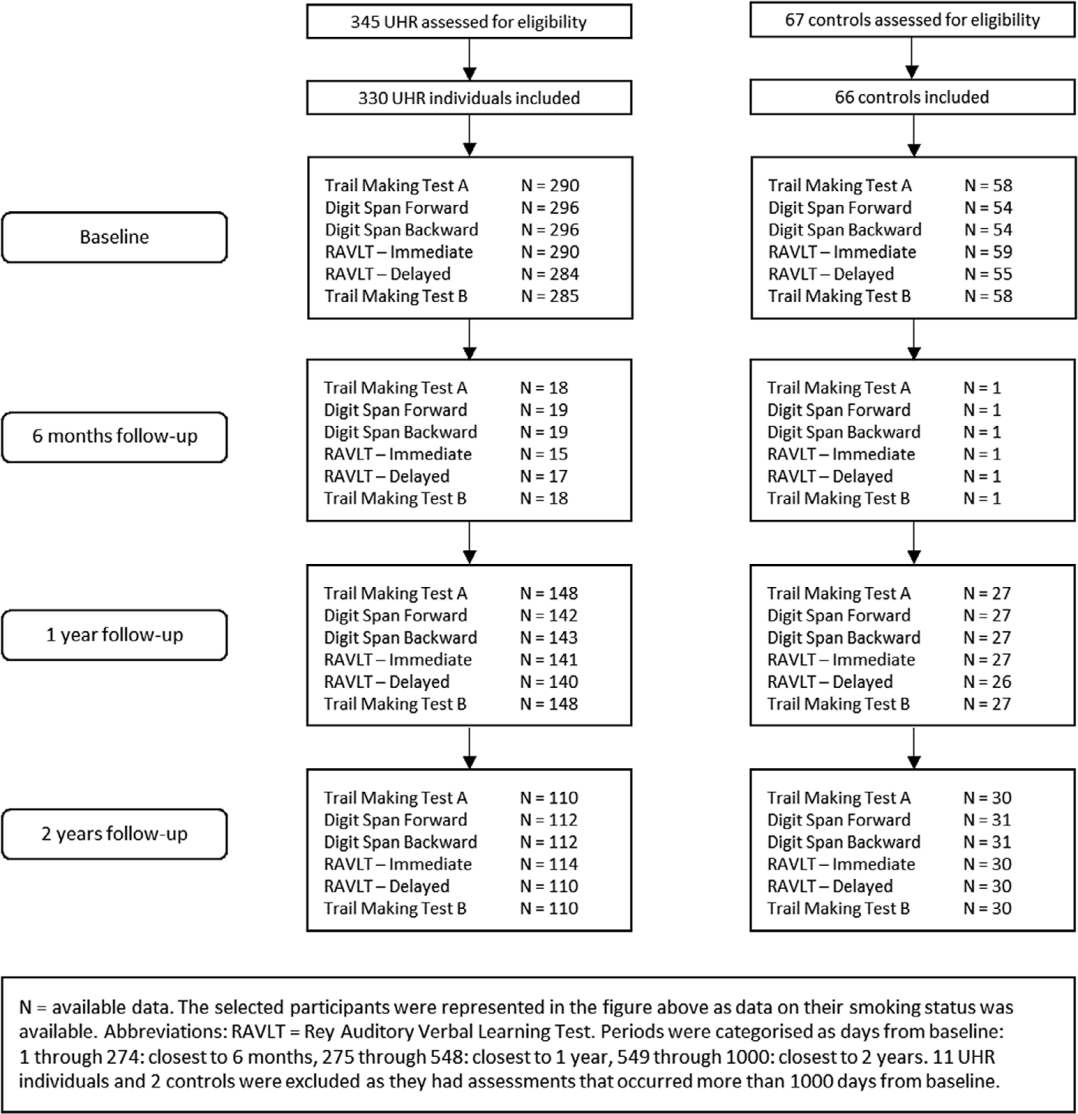
Available date per cognitive measurement over time in ultra-high risk (UHR) individuals and healthy controls.

In total, 345 UHR individuals and 67 healthy controls participated in the study (Figure 1). For the current analyses, only participants with data on smoking were included (330 UHR and 66 healthy controls). Sociodemographic features and clinical characteristics are presented in Table 1. In total, 11 UHR individuals and 2 healthy controls had assessments dates with extreme deviation (>1,000 days from baseline) on follow-up outcome variables. As we were interested in associations over approximately a 2-year period, these participants were excluded from the mixed model analysis. Data on cognitive performance scores were missing for a maximum of 58 (17.6%) UHR individuals and 23 (34.8%) healthy controls at baseline. See Supplementary Material S2 for detailed information regarding missing data on covariates and cognitive performance scores (Table 2).

**Table 1.** Demographic characteristics of smoking and nonsmoking ultra-high risk (UHR) individuals and controls.

	Nonsmoking UHR *N* = 155 (47%)	Smoking UHR *N* = 175 (53%)	*p* value	Nonsmoking controls *N* = 48 (72.7%)	Smoking controls *N* = 18 (27.3%)	*P* value
Male	77 (50%)	100 (57%)	0.186	26 (54%)	8 (44%)	0.482
Age	21.7 (4.8)	23.1 (5.0)	0.005	22.7 (3.7)	23.4 (5.1)	0.983
GAF (disability score)	57.5 (13.4)	53.8 (11.3)	0.015	85.0 (8.2)	84.7 (11.4)	0.828
Education (in years)	14.5 (3.1)	14.3 (3.0)	0.601	16.3 (2.9)	15.6 (2.2)	0.338
IQ	101.2 (17.7)	96.0 (16.2)	0.008	113.8 (8.9)	110.2 (17.0)	0.492
Current employment						
Student/paid work	92 (62%) *N* = 148	84 (52%) *N* = 162	0.067	41 (87.2%)	18 (100%)	0.112
Cannabis						
Ever used cannabis	75 (48%)	163 (94%) *N* = 173	<0.001	24 (50%)	17 (94.4%)	0.001
Current use of cannabis	17 (11%)	67 (39%) *N* = 173	<0.001	6 (23.1%)	12 (70.6%) *N* = 17	0.002
Trauma						
Total trauma score	9.3 (3.1)	9.8 (3.2)	0.167	7.0 (2.3)	6.7 (1.7)	0.843
CAARMS						
General symptoms	55.0 (29.0)	58.7 (29.3)	0.404	–	–	–
Positive symptoms	36.8 (20.0)	37.0 (19.7)	0.910	–	–	–
Negative symptoms	28.2 (18.5)	30.6 (18.6)	0.179	–	–	–
Psychiatric medication						
Use of antipsychotics	11 (9%) *N* = 124	15 (10%) *N* = 148	0.724	–	–	–
Use of antidepressants	40 (32%) *N* = 124	40 (27%) *N* = 148	0.346	–	–	–
Use of anxiolytics	12 (10%) *N* = 124	14 (9%) *N* = 148	0.951	–	–	–
No medication	75 (61%) *N* = 124	97 (66%) *N* = 148	0.389	–	–	–

Data are presented as *N* (%) or mean (standard deviation).

Abbreviations: CAARMS, Comprehensive Assessment of At-risk Mental State; GAF, Global Assessment of Functioning; IQ, estimated Intelligence Quotient.

**Table 2.** Multicross-sectional results from linear mixed models regarding smoking status and cognitive performance in UHR individuals and controls.

	UHR subjects (*N* = 330)			Controls (*N* = 66)		
Effects	Estimate	SE	*P*	Estimate	SE	*P*
Speed processing: Trail Making Test A^						
Intercept	34.435	2.052	<0.001	30.181	4.964	<0.001
Smoking	−0.443	1.100	0.687	−1.916	2.713	0.482
Attention/vigilance: Digit Span Forward						
Intercept	9.428	0.396	<0.001	10.216	0.991	<0.001
Smoking	0.146	0.184	0.429	−0.046	0.405	0.909
Working memory: Digit Span Backward						
Intercept	6.849	0.395	<0.001	5.884	1.034	<0.001
Smoking	0.266	0.203	0.191	−0.177	0.508	0.728
Verbal learning: RAVLT—immediate						
Intercept	46.844	1.742	<0.001	50.512	3.229	<0.001
Smoking	−0.888	0.820	0.279	−1.672	1.669	0.319
Verbal learning: RAVLT—delayed						
Intercept	9.144	0.529	<0.001	9.725	1.064	<0.001
Smoking	−0.089	0.269	0.742	0.778	0.566	0.173
Reasoning/problem solving: Trail Making Test B^						
Intercept	91.115	5.405	<0.001	60.261	7.262	<0.001
Smoking	−3.490	2.876	0.225	−0.904	3.591	0.802

The following fixed effects were added to the model: age + gender (model 1) + time. Subjects were added as random intercept and time was added as random slope.

Abbreviation: RAVLT = Rey Auditory Verbal Learning Test; SE, standard error.

### Baseline characteristics of smoking and nonsmoking individuals

At baseline, 175 (53%) UHR individuals and 18 (27%) controls reported daily smoking within the last 12 months. Smoking UHR individuals and controls smoked an average of 12.0 (SD = 8.7) and 9.7 (SD = 8.5) cigarettes per day, respectively. Baseline comparisons showed that smoking UHR subjects were significantly older than nonsmokers, reported more lifetime or current cannabis use, had lower GAF scores, and had lower IQ. Smoking control subjects significantly used more cannabis than nonsmoking controls, as listed in Table 1. In total, 35% of smoking individuals used psychiatric medication (of whom 10% used antipsychotics) compared to 40% of nonsmoking individuals (of whom 9% used antipsychotics). Baseline cognitive performance scores between smoking and nonsmoking UHR and controls are listed in Supplementary Material S3.

### Multi-cross-sectional associations between smoking behavior and cognitive performance

Mixed model analyses showed no significant differences between UHR individuals and controls who did or did not smoke on any of the cognitive performance scores, as listed in Table 3. In the second set of mixed model analyses, the number of cigarettes smoked per day was added as an independent variable instead of smoking status. These models revealed no significant associations between the number of cigarettes per day and cognitive performance scores (see Supplementary Material S4).

**Table 3.** Longitudinal results from linear mixed models regarding change in smoking status and change in cognitive performance in UHR.

Effects	Estimate	SE	p*
Speed processing: Trail Making Test A			
Intercept	−0.423	3.409	0.908
Start smoking	−4.475	2.306	0.153
Intercept	−5.276	5.900	0.373
Quit smoking	4.216	4.207	0.320
Attention/vigilance: Digit Span Forward			
Intercept	0.219	0.671	0.744
Start smoking	−0.644	0.525	0.224
Intercept	0.522	0.648	0.429
Quit smoking	0.447	0.450	0.331
Working memory: Digit Span Backward			
Intercept	1.312	0.878	0.140
Start smoking	−0.025	0.627	0.968
Intercept	0.188	0.842	0.824
Quit smoking	0.523	0.588	0.378
Verbal learning: RAVLT—immediate			
Intercept	4.156	2.651	0.119
Start smoking	−1.221	1.915	0.525
Intercept	2.496	2.654	0.350
Quit smoking	1.057	1.806	0.560
Verbal learning: RAVLT—delayed			
Intercept	1.878	1.138	0.134
Start smoking	−1.140	0.860	0.221
Intercept	0.865	1.085	0.427
Quit smoking	−0.454	0.746	0.544
Reasoning/problem solving: Trail Making Test B			
Intercept	−11.128	12.446	0.412
Start smoking	−13.126	9.420	0.170
Intercept	−7.688	15.009	0.652
Quit smoking	1.562	10.792	0.893

The following fixed effects were added to the model: age + gender (model 1) + time. Subjects were added as random intercept and time was added as random slope. Reference group: no smoking (vs. start) and continue smoking (vs. quit).

Abbreviation: RAVLT = Rey Auditory Verbal Learning Test.

### Longitudinal associations regarding change in smoking status and change in cognitive performance

Data on smoking behavior over time was available for 43% of UHR individuals and 46% of controls (Supplementary Material S5). From these, 84.8% of UHR individuals did not change their smoking behavior between two assessments: a total of 37.7% never smoked and 47.3% continued to smoke between assessments. Over time, 10.7% of UHR individuals quit and 6.4% started smoking.

In the second set of mixed model analyses, nonsmoking individuals over time (*N* = 106) were compared to individuals who started to smoke between baseline and 1-year follow-up, or between 1-year and 2-year follow-up (*N* = 18). No significant between-group differences regarding change in cognitive performance score were found. A second set was applied between individuals who continued to smoke over time (*N* = 130), compared to individuals who quit between baseline and 1-year follow-up, or between 1-year and 2-year follow-up (*N* = 27). No significant differences were found between cognitive performance scores of individuals who continued smoking and those who stopped smoking. See for full details Table 3. Concerning controls, 83.6% did not adjust smoking behavior over time. 67.2% (*N* = 41) never smoked and 16.4% (*N* = 10) continued to smoke. Of all controls with available smoking data, 8.2% (*N* = 5) quit as well did 8.2% (*N* = 5) initiated to smoke over time.

### Longitudinal association regarding change in the number of cigarettes and change in cognitive performance

Explorative analyses were conducted in 40.5% of UHR individuals for whom it was possible to calculate a change score in the number of cigarettes smoked per day between assessments (Supplementary Material S5a). No significant associations were found between change in the number of cigarettes smoked per day and change in any of the cognitive performance scores (Supplementary Material S6).

## Discussion

This study showed that in both UHR individuals and healthy controls, none of the tested cognitive domains (*speed of processing*, *attention/ vigilance*, *working memory*, *verbal learning,* or *reasoning/problem solving*) revealed multicross-sectional or longitudinal associations with (change) in smoking behavior across a two-year follow-up. Smoking prevalence was high (53%) in this sample of UHR individuals, compared to our control group (27.3%) and findings in the general population [37]. The cognitive performance of our sample was comparable to UHR samples from previous studies [14,38] and tended to be intermediary between individuals with a first episode and healthy individuals [39,40]. To the best of our knowledge, this is the first prospective study to evaluate the relationship between naturalistic smoking and cognitive functioning on different domains in UHR and healthy controls.

Our multicross-sectional findings are at odds with the findings of Gupta et al. [12] who reported better *visual learning,* higher *processing speed*, and improved *working memory* associated with chronic smoking in 35 UHR individuals. Nonetheless, the effect-sizes of correlations in their study were small (*r* = 0.33, *r* = 0.29, and *r* = 0.30, respectively). Furthermore, they defined smoking behavior differently (i.e., categorically) and different tasks were used to evaluate cognitive functions (except for the Trail Making Test A). More importantly, correlations between smoking and cognitive function were only corrected for IQ. The use of cannabis was not mentioned as a confounding variable, although the history of a substance dependence disorder in the prior 6 months was an exclusion criterion.

Two prior studies [41,42] in UHR evaluated smoking in relation to sensorimotor gating using prepulse inhibition (PPI), a biological measure proposed to be associated with cognitive functioning [43,44]. Cadenhead et al. [41] reported *greater* PPI—reflecting better sensorimotor gating—in chronic tobacco smoking UHR compared to nonsmoking UHR subjects. By contrast, De Koning et al. [42] did find a smoking x group interaction effect in the UHR group which was associated with lower prepulse inhibition. These contradictory results underline that further research is required to elucidate the impact of smoking on cognitive functioning as measured by cognitive batteries and biological measures in UHR.

Associations between smoking and worse cognitive functioning have been found repeatedly in patients with psychosis: a recent meta-analysis by Coustals et al. [10] evaluating 18 studies concluded that smoking patients had worse performance on certain cognitive tasks than nonsmoking patients. However, studies focusing on patients with a first-episode psychosis showed contradictory results. Some authors [45] reported worse global cognition in smokers, which is in line with findings in psychosis and the general population. Others [46–49] did not find any differences between smoking and nonsmoking FEP and some authors found cognitive-enhancing effects of nicotine [50]. To the best of our knowledge, no systematic review nor meta-analyses exist on this topic. In the general population, it has been shown that the longer people smoke (measured by pack-years), the higher the risk of cognitive deficits [51]. The lack of associations between smoking and cognitive functioning in the current study might be explained by the fact that our UHR subjects and controls were on average younger (22 years) than the subjects included in the meta-analysis of Coustals et al. (27–49 years) [10] and the general population study (56 years) [51]. Consequently, our UHR subjects might not have smoked for that many years and thus, not suffer yet from cumulative brain damage caused by smoking-induced oxidative stress, inflammation, and white matter lesion progression, which have been associated with cognitive decline [52,53].

An alternative explanation for the absence of an association between smoking and cognition in UHR might be that latent psychotic processes caused deterioration of cognitive performance in both smoking and nonsmoking UHR, covering a true smoking effect. However, the fact that we did not find any associations between smoking (and the number of cigarettes) and cognition in our control group argues against this explanation as this group did not suffer from illness-related symptoms. Additionally, one might state that in some individuals, the use of nicotine played an etiologic role [54,55] in crossing the threshold to UHR, while others met UHR criteria without any substance-related risk factors and might be more susceptible for (subclinical) and cognitive symptom severity.

A secondary finding of the current study was the fact that 10.3% of UHR individuals were able to quit smoking over a 1-year follow-up period. Cessation rates were almost twice as high compared to an UHR population studied by Ward et al. [56] (5.5% cessation) in which smoking cessation was evaluated over a longer follow-up period (2-year). Sustained quitting is associated with a shorter follow-up period [57] which might be a plausible explanation for higher cessation rates in the current study. In a FEP population, 3.9% quitted smoking during a 15-month follow-up period [58]. Together, these data suggest that early interventions focused on smoking cessation and relapse prevention in UHR are worthy of investigation. We did not find an association between smoking initiation (*N* = 19) or smoking cessation (*N* = 30) and change in any of the tested cognitive domains in these small subsamples. These results are in contrast with studies conducted in patients with psychosis and the general population, which found a positive relationship between smoking cessation and improvements of *processing speed* [11] and overall cognitive functioning [9], respectively. In the general population study [9], a longer time since smoking cessation was associated with higher differences in cognitive performance compared to current smokers. This may explain the lack of an association in our study as cognitive performance was evaluated after a 1-year interval of smoking cessation, compared to a 3-year interval in psychosis [11] and a 10-year interval in the general population [9]. Furthermore, the current study may not have been sufficiently powered to reach the statistic threshold as only 30 individuals were included in the change analysis for smoking cessation, compared to substantially larger samples in the general population (*N* = 602) [9] and in patients with psychosis (*N* = 517) [11].

Our results suggest that although the smoking prevalence in UHR is higher [59] and cognitive functioning is poorer compared to healthy controls [13], associations between smoking behavior and cognitive performance were not detected. This might indicate that smoking in UHR individuals is initiated for other reasons than (effective) alleviation of poorer cognitive functioning as suggested by the self-medication hypothesis [60]. A shared genetic and environmental architecture [61,62] between smoking and psychosis could partially explain why these UHR individuals already smoke so frequently and heavily.

### Strengths and limitations

The strengths of the current study lie in the prospective design together with the inclusion of a relatively large sample of UHR subjects (*N* = 330) and a healthy control group (*N* = 66). Furthermore, several sites across the world took part in this study, which increases its generalizability and importance for public health interventions. This study has also several limitations that should be acknowledged. First, participants were not asked or instructed about smoking behavior before they underwent the cognitive battery. Although smoking was not allowed during the assessment, potential acute pre-assessment effects of nicotine and/or withdrawal effects could have influenced cognitive function. Future studies should carefully consider smoking behavior when applying cognitive measurements. In addition, no data were available regarding levels of carbon monoxide or cotinine. Nevertheless, interviewer-reported questionnaires (such as the CIDI) were shown to produce accurate data regarding the validity of self-reported smoking behavior [63]. Secondly, the degree of tobacco use was administered using the CIDI [20], which has a scope of 12 months. Hence, smoking history (i.e., pack-years) was not assessed. Potential brain damage caused by chronic smoking was not taken into account. However, given the fact that our UHR population is relatively young, chronic smoking is less plausible. Third, assessments at 6 months were scarce in most centers due to differences in study design procedures. Also, a substantial number of participants were lost to 1- and 2-year follow-up. Fourth, in our explorative analyses evaluating change in smoking behavior and cognitive performance, our model was not able to calculate a *p* value of the estimate of the random slope and in some cases, no random intercept. However, sensitivity analyses were done without time as random slope which revealed similar results as the primary model. Lastly, the fact that only help-seeking individuals were included in the study may have led to a selection bias which limits the generalization of our findings.

## Conclusion

UHR individuals who smoked did not exhibit cognitive differences compared to nonsmoking UHR. However, the finding that one in every two UHR individuals daily uses tobacco represents a major health issue that demands priority in treatment. Comparisons with previous literature suggest that our UHR sample shows fewer cognitive impairments and higher smoking cessation rates compared to patients with FEP. Implications to promote smoking cessation in the UHR stage needs further investigation.

## Data Availability

Data are not publicly available.

## Supplementary Materials

To view supplementary material for this article, please visit http://dx.doi.org/10.1192/j.eurpsy.2021.2233. click here to view supplementary material
