# Impact of smoking behavior on cognitive functioning in persons at risk for psychosis and healthy controls: A longitudinal study – CORRIGENDUM

**DOI:** 10.1192/j.eurpsy.2022.10

**Published:** 2022-02-28

**Authors:** Heleen S. van der Heijden, Frederike Schirmbeck, Matthew J. Kempton, Mark van der Gaag, Kelly Allott, Barnaby Nelson, Stephan Ruhrmann, Lieuwe de Haan, Jentien M Vermeulen, Eu-Gei High Risk Study

**Keywords:** clinical high risk, cognition, corrigendum, nicotine, psychosis, smoking

This article was originally published with a spelling error in author Kelly Allott’s name. This error has now been corrected and this corrigendum published.
