# Supplementary material for: Impact of smoking Behavior on cognitive functioning in persons at risk for psychosis and healthy controls: A longitudinal study
Source: Eur Psychiatry. 2021 Sep 21;64(1):e60. doi: 10.1192/j.eurpsy.2021.2233 (PMC8516743; doi:10.1192/j.eurpsy.2021.2233)
Supplement: Supplementary file 1 [file S0924933821022331sup001.docx]

APPENDIX

This appendix formed part of the original submission.

Supplement to:

*Impact of Smoking Behavior on Cognitive Functioning in Persons at Risk for Psychosis and Healthy Controls: A Longitudinal Study*

Van der Heijden HS, Schirmbeck NF, de Haan L and Vermeulen JM. et al.

Index:

- Supplement 1: UHR individuals and controls with follow-up assessment by site page 2
- Supplement 2: UHR individuals and controls with missing data at baseline page 2
- Supplement 3: Baseline cognitive performance scores in smoking and page 3
  non-smoking UHR individuals and controls
- Supplement 4: Multi-cross-sectional results from linear mixed models regarding page 3
  the number of cigarettes and cognitive performance scores in UHR and controls
- Supplement 5: Available data on change in smoking behaviour per subgroup page 4
- Supplement 6: Longitudinal results from linear mixed models regarding page 5
  change in the number of cigarettes and change in cognitive functioning
- List of EUGEI authors not mentioned as main authors page 6

Supplement 1: Participants with follow-up assessment by site

|  | *Baseline* | | *6 months* | | *1 year* | | *2 years* | |
| --- | --- | --- | --- | --- | --- | --- | --- | --- |
| *Site* | UHR | Controls | UHR | Controls | UHR | Controls | UHR | Controls |
| London | 97 | 38 | 22 | 17 | 40 | 20 | 39 | 25 |
| Amsterdam | 14 | 5 | 0 | 0 | 5 | 5 | 2 | 5 |
| Den Haag | 63 | 4 | 0 | 0 | 42 | 4 | 42 | 4 |
| Vienna | 12 | 0 | 0 | 0 | 2 | 0 | 1 | 0 |
| Basel | 19 | 0 | 1 | 0 | 14 | 0 | 4 | 0 |
| Cologne | 15 | 0 | 1 | 0 | 9 | 0 | 6 | 0 |
| Melbourne | 35 | 19 | 6 | 0 | 6 | 7 | 3 | 0 |
| Copenhagen | 18 | 0 | 0 | 0 | 12 | 0 | 4 | 0 |
| Paris | 19 | 0 | 5 | 0 | 7 | 0 | 0 | 0 |
| Barcelona | 23 | 0 | 0 | 0 | 14 | 0 | 10 | 0 |
| Sao Paulo | 15 | 0 | 1 | 0 | 1 | 0 | 2 | 0 |
| Missing | 0 | 0 | 8 | 0 | 25 | 0 | 15 | 0 |
| Total | 330* | 66* | 44 | 15 | 177 | 36 | 127 | 34 |
| In total, 330 UHR and 66 controls were included as smoking data and information on at least one cognitive measurement was available at baseline. Periods were categorised as days from baseline: 1 through 274: closest to 6 months, 275 through 548: closest to 1 year, 549 through 1000: closest to 2 years. 11 UHR individuals and 2 controls were excluded as they had assessments that occurred more than 1000 days from baseline. | | | | | | | | |

Supplement 2: UHR individuals and controls with missing data at baseline

| *Variable* | Missing at baseline | |
| --- | --- | --- |
|  | UHR individuals | Controls |
| GAF | 7 (2.1%) | 1 (1.5%) |
| Work | 20 (6.1%) | 1 (1.5%) |
| Education in years | 30 (9.1%) | 1 (1.5%) |
| Estimated Intelligence Quotient | 27 (8.2%) | 3 (4.5%) |
| Cannabis (current use) | 2 (0.6%) | 23 (34.8%) |
| Cannabis (ever used) | 2 (0.6%) | 0 (0%) |
| Trauma total score | 22 (6.7%) | 1 (1.5%) |
| Medication | 58 (17.6%) | n.a. |
| CAARMS - general symptoms | 31 (9.4%) | n.a. |
| CAARMS - positive symptoms | 8 (2.4%) | n.a. |
| CAARMS - negative symptoms | 14 (4.2%) | n.a |
| Trail Making Test A | 40 (12.1%) | 8 (12.1%) |
| Digit Span Forward | 34 (10.3%) | 12 (18.2%) |
| Digit Span Backward | 34 (10.3%) | 12 (18.2%) |
| RAVLT - Immediate | 40 (12.1%) | 7 (10.6%) |
| RAVLT - Delayed | 46 (13.9%) | 11 (16.7%) |
| Trail Making Test B | 45 (13.6%) | 8 (12.1%) |
| Abbreviations: GAF = Global Assessment of Functioning. CAARMS= Comprehensive Assessment of At-risk Mental State. Abbreviations: RAVLT = Rey Auditory Verbal Learning Test. | | |

Supplement 3: Baseline cognitive performance scores in smoking and non-smoking participants

|  | | UHR (N= 330) | | Controls (N=66) | |
| --- | --- | --- | --- | --- | --- |
| *Cognitive domain* | Cognitive task | Non-smoking | Smoking | Non-smoking | Smoking |
| Speed processing | Trail Making Test part A^ | 30.0 (11.1) | 30.1 (13.0) | 27.8 (14.6) | 24.6 (7.3) |
| Attention/ vigilance | Digit Span Forward (0-16) | 9.3 (2.4) | 9.7 (2.1) | 10.6 (2.4) | 10.7 (2.2) |
| Working memory | Digit Span Backward (0-14) | 6.4 (2.1) | 6.8 (2.3) | 7.5 (2.7) | 7.4 (2.4) |
| Verbal learning | RAVLT – immediate (0-75) | 52.0 (10.1) | 50.6 (9.9) | 56.1 (9.5) | 56.9 (6.0) |
|  | RAVLT – delayed (0-15) | 10.8 (2.8) | 10.4 (3.2) | 11.1 (3.4) | 12.1 (3.6) |
| Reasoning/  problem solving | Trail Making Test part B^ | 73.5 (31.3) | 72.0 (28.3) | 57.4 (20.7) | 54.3 (14.5) |
| Data are in mean (SD). RAVLT = Auditory Verbal Learning Test. ^Higher scores indicate greater impairment. | | | | | |

Supplement 4: Multi-cross-sectional results from linear mixed models regarding the number of cigarettes and cognitive performance scores in UHR individuals and controls

|  | UHR | | | Controls | | |
| --- | --- | --- | --- | --- | --- | --- |
| *Effects* | *Estimate* | *SE* | *p* | *Estimate* | *SE* | *p* |
| Speed processing: Trail Making Test A | | | | | | |
| Intercept | 33.971 | 2.046 | <0.001 | 29.743 | 5.055 | <0.001 |
| Cigarettes | 0.051 | 0.064 | 0.423 | 0.018 | 0.244 | 0.942 |
| Attention/ vigilance: Digit Span Forward | | | | | | |
| Intercept | 9.462 | 0.395 | <0.001 | 10.223 | 0.999 | <0.001 |
| Cigarettes | 0.005 | 0.011 | 0.620 | -0.003 | 0.037 | 0.942 |
| Working memory: Digit Span Backward | | | | | | |
| Intercept | 6.932 | 0.391 | <0.001 | 5.942 | 1.045 | <0.001 |
| Cigarettes | 0.005 | 0.011 | 0.669 | -0.023 | 0.047 | 0.635 |
| Verbal learning: AVLT – immediate | | | | | | |
| Intercept | 46.340 | 1.717 | <0.001 | 49.962 | 3.238 | <0.001 |
| Cigarettes | -0.010 | 0.047 | 0.831 | 0.087 | 0.151 | 0.566 |
| Verbal learning: AVLT – delayed | | | | | | |
| Intercept | 9.123 | 0.520 | <0.001 | 9.821 | 1.103 | <0.001 |
| Cigarettes | -0.002 | 0.015 | 0.921 | 0.017 | 0.058 | 0.773 |
| Reasoning/ problem solving: Trail Making Test B | | | | | | |
| Intercept | 89.859 | 5.449 | <0.001 | 58.822 | 7.338 | <0.001 |
| Cigarettes | -0.046 | 0.167 | 0.783 | 0.343 | 0.343 | 0.320 |
| AVLT = Auditory Verbal Learning Test. SE = Standard Error. The following fixed effects were added to the model: age + gender (model 1) + time. Subjects were added as random intercept and time was added as random slope. | | | | | | |

Supplement 5a: Available data on change in smoking behaviour over time in UHR individuals

|  | *Baseline – 1 year* | *1 year – 2 years* |
| --- | --- | --- |
| SUBGROUP | | |
| No smoker | 63 | 43 |
| Continue smoker | 84 | 46 |
| Quit smoker | 18 | 9 |
| Start smoker | 11 | 7 |
| Total | 176 | 105 |
| CIGARETTES | | |
| Change in cigarettes | 79 | 53 |
| No change | 85 | 50 |
| Total | 164 | 103 |
| N = available data. | | |

Supplement 5b: Available data on change in smoking behaviour per subgroup over time in controls

|  | *Baseline – 1 year* | *1 year – 2 years* |
| --- | --- | --- |
| SUBGROUP | | |
| No smoker | 24 | 17 |
| Continue smoker | 6 | 4 |
| Quit smoker | 4 | 1 |
| Start smoker | 1 | 4 |
| Total | 35 | 26 |
| CIGARETTES | | |
| Change in cigarettes | 9 | 8 |
| No change | 26 | 18 |
| Total | 35 | 26 |
| N = available data. | | |

Supplement 6: Longitudinal results from linear mixed models regarding change in the number of cigarettes
and change on cognitive performance in UHR individuals

| Effects | Estimate | SE | p |
| --- | --- | --- | --- |
| Speed processing: Trail Making Test A | | | |
| Intercept | -3.453 | 3.418 | 0.313 |
| Cigarettes | -0.015 | 0.125 | 0.907 |
| Attention/ vigilance: Digit Span Forward | | | |
| Intercept | 0.412 | 0.471 | 0.384 |
| Cigarettes | -0.016 | 0.017 | 0.352 |
| Working memory: Digit Span Backward | | | |
| Intercept | 0.738 | 0.567 | 0.197 |
| Cigarettes | -0.002 | 0.020 | 0.929 |
| Verbal learning: AVLT – immediate | | | |
| Intercept | 3.436 | 1.904§ | 0.073 |
| Cigarettes | 0.019 | 0.068 | 0.778 |
| Verbal learning: AVLT – delayed | | | |
| Intercept | 1.119 | 0.784§ | 0.129 |
| Cigarettes | 0.019 | 0.028 | 0.509 |
| Reasoning/ problem solving: Trail Making Test B | | | |
| Intercept | -13.454 | 13.619 | 0.384 |
| Cigarettes | 0.102 | 0.397 | 0.800 |
| RAVLT = Rey Auditory Verbal Learning Test. SE = Standard Error. The following fixed effects were added to the model: age + gender (model 1) + time. Subjects were added as random intercept and time was added as random slope. | | | |

**EU-GEI authors not mentioned as main author:**

Mathilde Antoniades^1^, Sara Pisani^1^, Stefania Tognin^1^, Gemma Modinos^1^, Emily P Hedges^1^, Eva Velthorst^2,3^, Tamar C. Kraan^2^, Daniella S. van Dam^2^, Nadine Burger^4^, Patrick McGorry^5^, G Paul Amminger^5^, Christos Pantelis^6^, Athena Politi^5^, Joanne Goodall^7^, Stefan Borgwardt^7^, Erich Studerus^7^, Ary Gadelha^8^, Elisa Brietzke^9^, Graccielle Asevedo^8^, Elson Asevedo^8^, Andre Zugman^8^, Tecelli Domínguez-Martínez^10^, Manel Monsonet^11^, Lídia Hinojosa^11^, Anna Racioppi^11^, Thomas R. Kwapil^12^, Mathilde Kazes^13,^ Claire Daban^13^, Julie Bourgin^13^, Olivier Gay^13^, Célia Mam-Lam-Fook^13^, Marie-Odile Krebs^13^, Dorte Nordholm^14^, Lasse Randers^14^, Kristine Krakauer^14^, Louise Birkendal Glenthøj^14^, Birte Glenthøj^15^, Dominika Gebhard^16^, Julia Arnhold^17^, Joachim Klosterkötter^16^, Iris Lasser^18,^ Bernadette Winklbaur^18^, Philippe A Delespaul^19,20^

Affiliations:

1. Department of Psychosis Studies, Institute of Psychiatry, Psychology & Neuroscience, King's College London, London, United Kingdom.
2. Amsterdam UMC, Department of Psychiatry, Amsterdam, the Netherlands
3. Icahn School of Medicine at Mount Sinai, department of Psychiatry, New York, United States.
4. Parnassia Psychiatric Institute, Department of Psychosis Research, The Hague, The Netherlands.
5. Centre for Youth Mental Health, University of Melbourne, Parkville, Victoria, Australia.
6. Melbourne Neuropsychiatry Centre, University of Melbourne & Melbourne Health, Carlton South, Australia
7. Medical Faculty, University of Basel, Basel, Switzerland
8. LiNC - Lab Interdisciplinar Neurociências Clínicas, Depto Psiquiatria, Escola Paulista de Medicina, Universidade Federal de São Paulo – UNIFESP, São Paulo, Brazil
9. Depto Psiquiatria, Escola Paulista de Medicina, Universidade Federal de São Paulo
10. Global Mental Health Research Center, Directorate of Epidemiological and Psychosocial Research, ‘Ramón de la Fuente Muñiz’ National Institute of Psychiatry, Mexico.
11. Departament de Psicologia Clínica i de la Salut (Universitat Autònoma de Barcelona).
12. Department of Psychology, University of Illinois at Urbana-Champaign (USA).
13. University Paris Descartes, Hôpital Sainte-Anne, C’JAAD, Service Hospitalo-Universitaire, Inserm U894, Institut de Psychiatrie (CNRS 3557) Paris, France
14. Mental Health Center Copenhagen and Center for Clinical Intervention and Neuropsychiatric Schizophrenia Research, CINS, Mental Health Center Glostrup, Mental Health Services in the Capital Region of Copenhagen, University of Copenhagen.
15. Centre for Neuropsychiatric Schizophrenia Research (CNSR) & Centre for Clinical Intervention and Neuropsychiatric Schizophrenia Research (CINS), Mental Health Centre Glostrup, University of Copenhagen, Glostrup, Denmark
16. Department of Psychiatry and Psychotherapy, Faculty of Medicine and University Hospital, University of Cologne, Cologne, German
17. Psyberlin, Berlin, Germany.
18. Department of Psychiatry and Psychotherapy, Medical University of Vienna, Austria
19. Department of Psychiatry and Neuropsychology, School for Mental Health and Neuroscience, Maastricht University Medical Centre, Maastricht, The Netherlands
20. Mondriaan Mental health Trust, P.O. Box 4436 CX Heerlen, The Netherlands
